# The Mechanism of Rac1 in Regulating HCC Cell Glycolysis Which Provides Underlying Therapeutic Target for HCC Therapy

**DOI:** 10.1155/2022/7319641

**Published:** 2022-07-06

**Authors:** Yin-Xiang Ren, Xiao-Bin Li, Wei Liu, Xu-Guang Yang, Xin Liu, Yu Luo

**Affiliations:** ^1^School of Basic Medical Sciences, Lanzhou University, Lanzhou 730000, China; ^2^School of Chinese Materia Medica, Beijing University of Chinese Medicine, Beijing 100029, China

## Abstract

**Aim:**

To explore the role of Rac1 on sorafenib resistance in hepatocellular carcinoma.

**Methods:**

CCK-8, wound healing assay, Transwell, and cell cycle assay were used to detect the tumor cells development. Cell viability was assessed by MTT. The glycolytic pathway was revealed by cellular metabolism assays.

**Result:**

We recovered that Rac1 upregulation was related to HCC patients' poorer prognosis. Forced expression of Rac1 promoted cell development and sorafenib chemoresistance in HCC cells. Rac1 inhibitor EHop-016 and sorafenib combination markedly prevented cell viability, G2/M phase cycle arrest, and apoptosis than single therapy. Furthermore, combination therapy decreased glycolysis in HCC cells. In vivo, the tumor growth was significantly prevented by combination therapy single therapy.

**Conclusion:**

Our research declares that Rac1 inhibition could block sorafenib resistance in HCC by decreasing glycolysis, which would provide an underlying target for HCC therapy.

## 1. Introduction

Hepatocellular carcinoma (HCC) is one of the most common and most invasive malignant tumors of the digestive system. Globally, the incidence of HCC ranks sixth among all malignant diseases [1, 2]. Due to the high HBV infection rate in China, about 50% of the world's HCC occurs in China [3, 4]. Due to the atypical symptoms of HCC in the early stage, the patient has lost the best opportunity for operation [5].

Sorafenib, as a first-line treatment for HCC, could effectively ameliorate the prognosis of HCC patients [6]. Sorafenib inhibits the proliferation of tumor cells by inhibiting Raf/MEK/ERK and PI3K/AKT/mTOR signaling pathways, thus inhibiting the progression of liver cancer [7–9]. In addition, sorafenib also has the ability to inhibit the VEGF receptor and PDGF receptor, further block tumor angiogenesis, and thus indirectly inhibit tumor growth [10, 11]. Sorafenib could prolong the survival time of patients with advanced HCC for 3 months, but most patients will develop sorafenib resistance after taking the drug [12]. Therefore, in order to effectively prolong the survival time of patients with advanced HCC, sorafenib resistance is an urgent problem to be solved.

Ras-associated C3 botulinum toxin subunit 1 (Rac1) is a classical affiliate of Ras superfamily Rho subfamily Rho GTP enzymes, which is closely related to a variety of physiological and biochemical activities of cells [13, 14]. At present, more and more evidences show that the abnormal activity and expression of Rac1 are closely related to tumorigenesis, survival, metastasis, antiapoptosis, drug resistance, and other tumor characteristics [15, 16]. The increase of Rac1 activity or expression caused by gene mutation or other factors can promote the occurrence, development, metastasis, and invasion of tumor, resulting in poor prognosis of patients [17]. Meanwhile, some mechanisms of the function of Rac1 in drug resistance and liver cancer were discussed. For example, it has been reported that Rac1 activates the nonoxidative pentose phosphate pathway to induce chemoresistance of breast cancer [18]. Rac1 is enhanced in hepatocellular carcinoma samples [19]. miR-365 and miR-194 modulate liver cancer stem cells via the RAC1 pathway [20, 21]. However, the function of Rac1 in the modulation of sorafenib resistance and glycolysis remains unclear. Specific inhibitors or gene knockout to inhibit the activity or expression of Rac1 can inhibit tumor invasion, metastasis, and other malignant behavior. The abnormal expression of Rac1 is also associated with tumor. The poor early surgical prognosis of some patients with nonsmall cell lung cancer is related to the Rac1 upregulation, which may be related to the fact that the high expression of Rac1 in nonsmall cell lung cancer tumor stem cells can enhance the malignant behavior of tumor cells [22]. This situation also exists in other tumors, such as hematological diseases, the upregulated expression of Rac1 could promote the occurrence of stem cell leukemia/lymphoma syndrome leukemia driven by FGFR1 and has correlation with lymph node metastasis, TNM stage, and poor differentiation [23]. At present, specific Rac1 inhibitors (such as EHop-016) have been found to have antitumor effect [24]. In previous research, Rac1 inhibition could effectively alleviate chemoresistance. At present, the function of Rac1 in chemoresistance of HCC has not been demonstrated.

Here, we revealed the correlation between Rac1 and the prognosis of HCC patients. Furthermore, we explored the function of Rac1 in HCC development and chemoresistance. We also detected the effectiveness and the associated mechanisms of combination therapy of sorafenib and the Rac1 inhibitor for HCC, which would provide the experimental basis for clinical treatment.

## 2. Material and Methods

### 2.1. Clinical Samples

Tumor tissues and adjacent normal tissues were collected. The adjacent tissue was taken 1 cm away from the tumor. The patient was not treated with chemotherapy and radiotherapy before operation. All the adjacent tissues were evaluated and confirmed by 2 pathologists. The study was approved by the Ethics Committee of Lanzhou University and carried out after the patient signed an informed consent form.

### 2.2. Cell Culture and Treatment

HCC cell lines were purchased from the Shanghai Institute of Life Sciences, Chinese Academy of Sciences. The cells were cultured in the ROMI-1640 medium containing 10% fetal bovine serum, 100 U/mL penicillin, and streptomycin in a 5% CO_2_ incubator at 37°C.

The cells were seeded in 6-well plates, and the plasmids were transfected into cells by Lipofectamine 3000. The transfection process was referred to the operation instructions provided by the kit.

### 2.3. Western Blot

Cells were collected and treated with RIPA lysate. Total protein was extracted. Protein concentration was explored by the BCA kit. Protein was separated by SDS-PAGE gel and transferred to the nitrocellulose membrane. The transfer membrane was blocked with a blocking solution containing 5% bovine serum albumin for 2 h, and the primary antibody was kept at 4°C overnight. Secondary antibody was kept at room temperature for 1 h. Finally, the enhanced chemiluminescence substrate reaction kit (Thermo Scientific product) was used to analyze the gray value of each protein band.

### 2.4. MTT Assay

5 × 10^3^ HCC cells/wells were seeded in a 96-well plate. After the cells grew to the bottom of the culture plate, they were treated with different concentrations of sorafenib (0, 1, 5, 10, and 20 *μ*mol/mL) for 24 h [25]. The IC50 of sorafenib was 12.05 and 11.65 *μ*mol/mL in hep3B and Huh7 cells. Then, the cells were incubated with MTT (5 mg/mL) for another 4 h. After removing the supernatant, 150 *μ*L DMSO was added into each well, and the crystal was fully dissolved after shaking for 10 min. The *D* value of each hole was detected at the wavelength of 490 nm, and the cell survival rate was calculated.

### 2.5. Flow Cytometry for Apoptosis

Cells were transferred to a 6-well plate and treated with sorafenib at the concentration of (0, 1, 2.5, 5, and 10 *μ*M) for 24 h. The cells were taken out and digested with trypsin. All cells (including cells in the supernatant) in each well were collected. Apoptosis dye PI 5 *μ*L / tube and FITC 10 *μ*L / tube were added and incubated in dark at 4°C for 20 min. Cell apoptosis data were analyzed on the computer.

### 2.6. Transwell Invasion Assay

Transwell invasion experiment was used. HCC cells were digested with trypsin. The cells were resuspended in RPMI-1640 containing 2% FBS and counted. 200 *μ*l cell suspension (5 × 104 cells/100 *μ*l) was placed in the upper chamber, and 20% FBS culture medium containing drugs was added in the lower chamber. After 48 h, the Transwell chamber was removed, and the culture medium in the chamber was discarded. After 3 times of precooling PBS washing, the cells were fixed with precooling methanol for 20 min and stained with 0.01% crystal violet for 20 min. PBS was used to clean the cell chamber, and cotton swabs were used to gently wipe the upper unmigrated cells. Five visual fields (×200) were randomly selected under the microscope to count the number of invasion cells through the membrane and take the average value.

### 2.7. CCK-8 Assay

HCC cells in good growth condition were digested with trypsin and resuspended with 5% FBS medium (5 × 10^4^/ml). The cells were inoculated into a 96-well culture plate and incubated overnight. The old culture medium was discarded. 100 *μ*L of the CCK8 reagent and DMEM1: 9 mixture was added to cells and incubated at 37°C for 2 h. The absorbance (A) of the liquid was measured at 450 nm using an enzyme labelling instrument.

## 3. Results

### 3.1. Rac1 Is Connected with Poor Prognosis in HCC Patients

**Figure 1:**
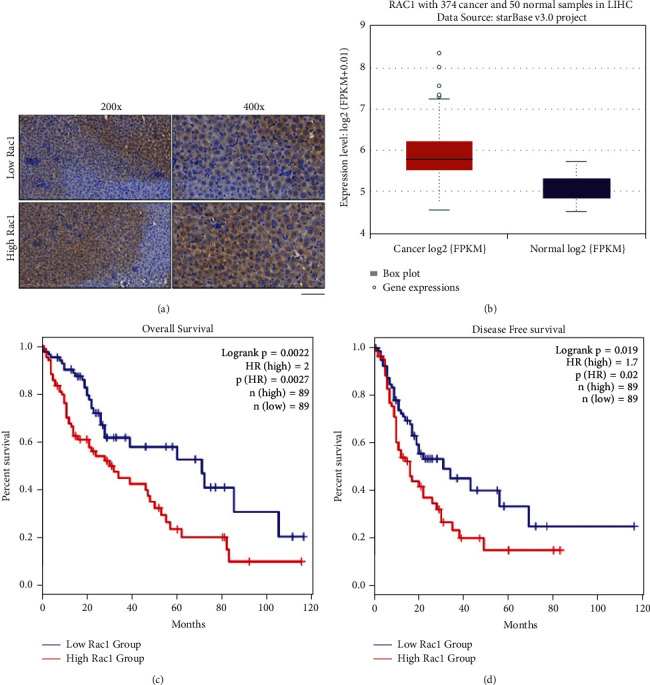
Rac1 expression and prognostic evaluation in HCC patient samples. (a). IHC detection of Rac1 expression in HCC patient samples. Scale bar = 100 *μ*m. (b). The expression of Rac1 in cancer and normal samples (Figures (c)&(d)). Survival analysis with the log-rank test evaluating the overall survival (OS) and disease-free survival (DFS) in patients expressing high or low levels of Rac1.

In order to explore the function of Rac1, we collected HCC patients' tumor tissues and detected the level of Rac1 (Figure 1(a)). Pan-cancer data showed that Rac1 was upregulated in 374 HCC patients (Figure 1(b)). Furthermore, the high level of Rac1 patients performed poor prognosis than the low level of Rac1 patients (Figures 1(c) and 1(d)).

### 3.2. Rac1 Promotes HCC Cell Development and Progression

**Figure 2:**
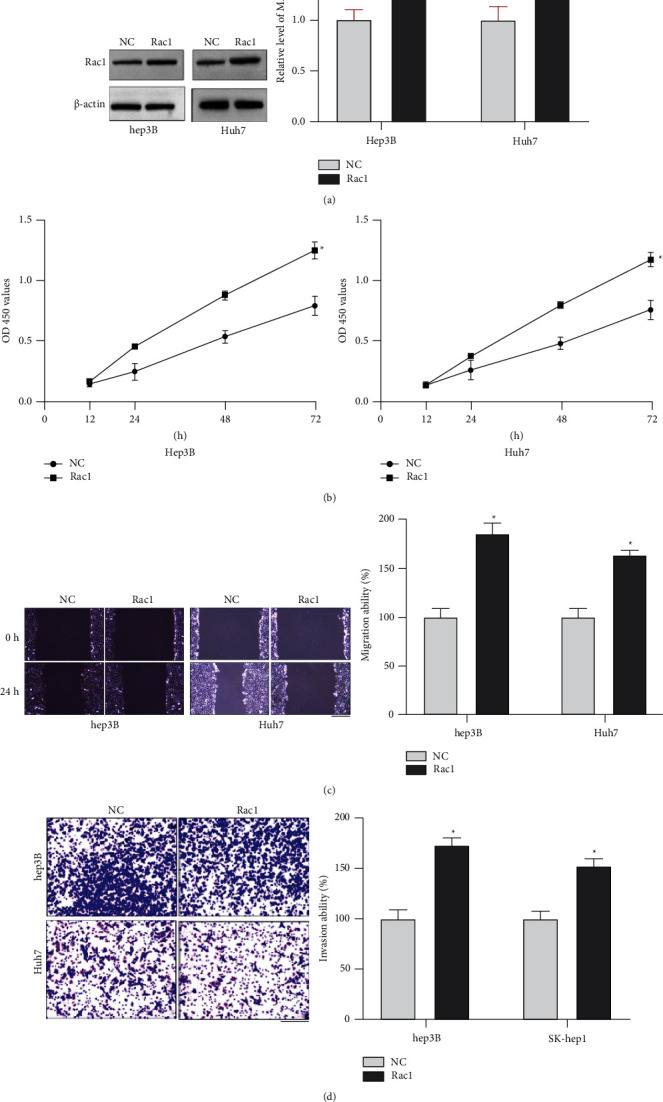
Effects of Rac1 on the proliferation and migration of HCC cells. (a). Western blot analysis of Rac1 expression in HCC cells at 48 h after transfection with NC or Rac1. (b). The CCK-8 assay was performed at 0, 12, 24, and 48 h after Rac1 transfection. (c). Representative images and quantitative analysis of the results from the wound healing assay. Scale bar = 100 *μ*m. (d). Representative images and quantitative analysis of the results from the Transwell invasion assay. Scale bar = 100 *μ*m. ^*∗*^*P* < 0.05. Statistical differences were analyzed using Student's *t*-tests. Error bars represent SEM from triplicate experiments.

Then, we constructed the plasmid for overexpression of Rac1, and we assessed the protein level of Rac1 in hep3B and Huh7 cells after Rac1 transfection. The expression pf Rac1 was increased in HCC cells (Figure 2(a)). The CCK-8 assay was used to detect the cell proliferation ability in HCC cells, and Rac1 promoted proliferation ability in hep3B and Huh7 cells (Figure 2(b)). The wound healing assay revealed that Rac1 increased migration ability in HCC cells (Figure 2(c)). The cell invasion ability was explored by the Transwell invasion assay, and Rac1 induced cell invasion in HCC cells (Figure 2(d)). In summary, Rac1 promoted HCC cell development and progression.

### 3.3. Silencing of Rac1 Prevents Proliferation, Migration, and Invasion Ability in HCC Cells

**Figure 3:**
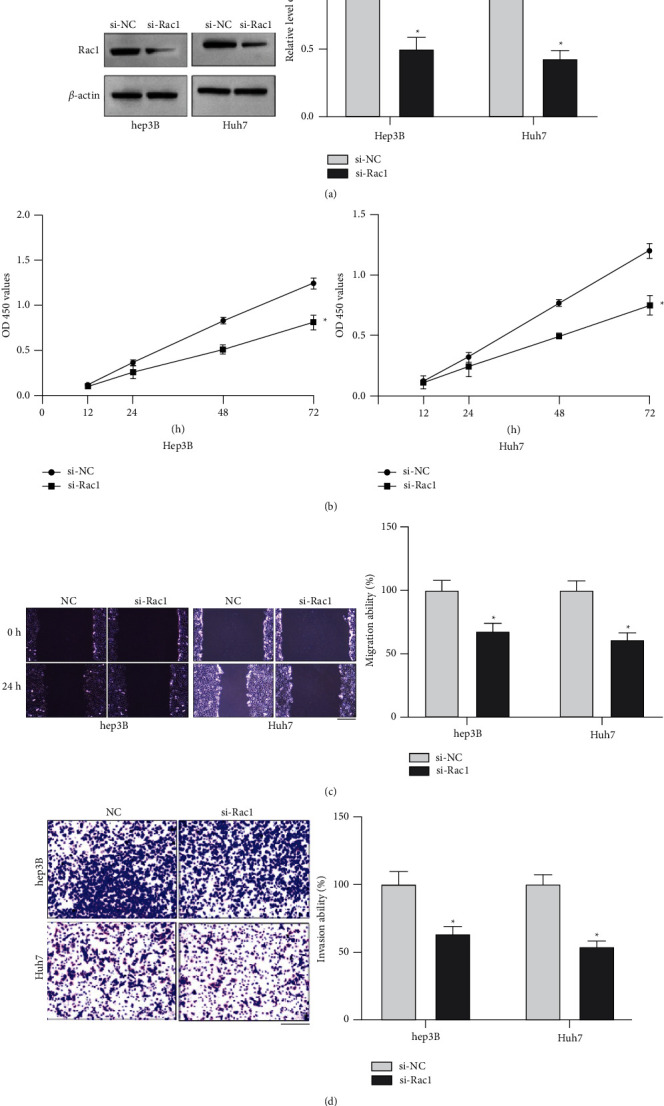
Effects of Rac1 on the proliferation and migration of HCC cells. (a). Western blot analysis of Rac1 expression in HCC cells at 48 h after transfection with si-NC or si-Rac1. (b). The CCK-8 assay was performed at 0, 12, 24, and 48 h after si-Rac1 transfection. (c). Representative images and quantitative analysis of the results from the wound healing assay. Scale bar = 100 *μ*m. (d). Representative images and quantitative analysis of the results from the Transwell invasion assay. Scale bar = 100 *μ*m. ^*∗*^*P* < 0.05. Statistical differences were analyzed using Student's *t*-tests. Error bars represent SEM from triplicate experiments.

SiRNA was constructed to inhibit the expression level of Rac1, and Rac1 downregulation was found by the Western blot assay (Figure 3(a)). CCK-8 assay results performed the decreased proliferation ability in HCC cells after si-Rac1 transfection (Figure 3(b)). The wound healing assay and Transwell invasion assay were used to detect the migration and invasion ability in HCC cells. The blocked migration and invasion ability were found in si-Rac1 transfected cells (Figures 3(c) and 3(d)). Taken together, silencing of Rac1 blocked HCC cell development.

### 3.4. Rac1 Affords Sorafenib Resistance to HCC In Vitro

**Figure 4:**
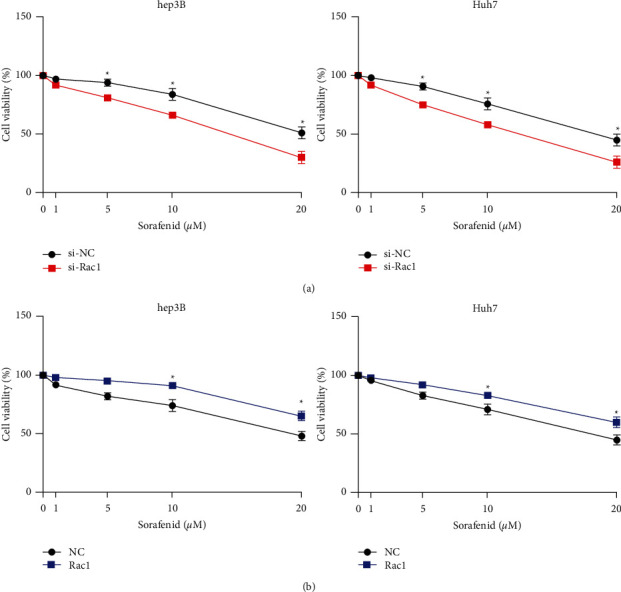
Correlation between RAC1 and Sorafenib resistance in HCC cells. (a). Chemoresistance was measured using the MTS assay in HCC cells with si-Rac1 transfected. (b). Chemoresistance was determined using the MTS assay in HCC cells with Rac1 transfected. ^*∗*^*P* < 0.05. Statistical differences were analyzed using Student's *t*-tests. Error bars represent SEM from triplicate experiments.

Sorafenib is an oral multikinase inhibitor which could inhibit angiogenesis and tumor development. Other studies have reported that sorafenib can also target signal transduction pathways, including apoptosis and cell cycle-related pathways. Drug resistance is the main reason for the limitation of sorafenib application, and its mechanism is complex and has not been fully revealed. At present, there was no research on the relationship between Rac1 and chemoresistance in HCC. We explored the effect of sorafenib on si-Rac1 or Rac1-transfected HCC cells using the MTT assay. Si-Rac1 prevented sorafenib resistance of hep3B and Huh7 cells than si-NC transfection (Figure 4(a)). Oppositely, Rac1 induced the sorafenib resistance in HCC cells than NC- transfected cells (Figure 4(b)).

### 3.5. Sorafenib Combination with the Rac1 Inhibitor Inhibits Chemoresistance to Sorafenib in HCC Cells

**Figure 5:**
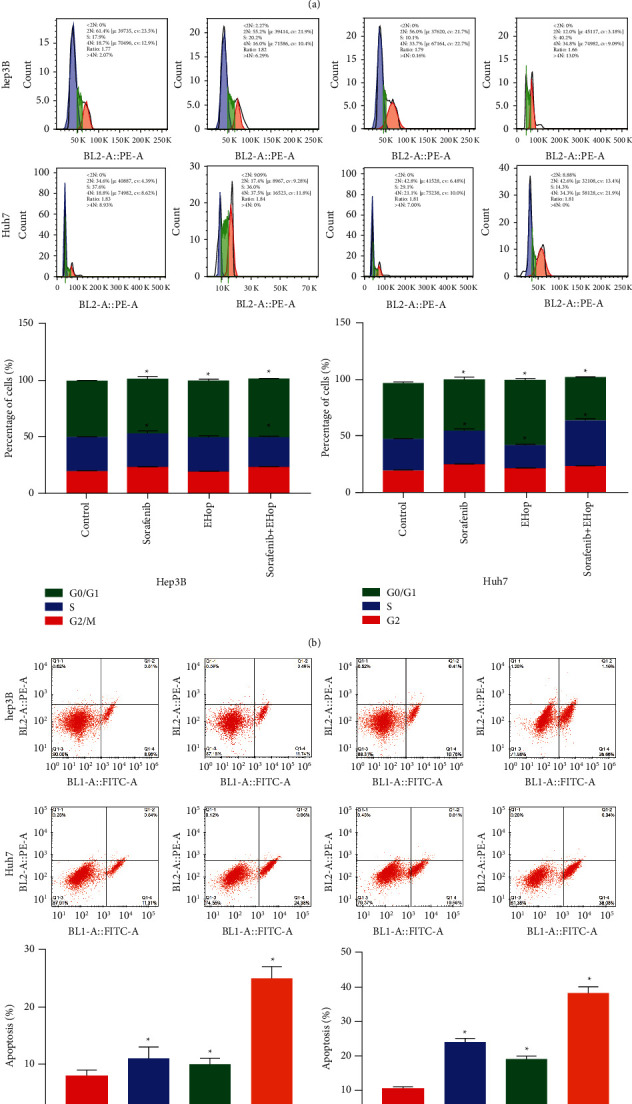
Antitumor effects of combination therapy of Sorafenib and the RAC1 inhibitor in HCC cells. (a). Cell viability was evaluated at 48 h after Sorafenib and EHop-016 treatment. (b). Cell cycle analysis using flow cytometry in HCC cells treated with the indicated concentration of Sorafenib, EHop-016, or combination therapy for 24 h. (c). The apoptotic rates of HCC cells treated with the indicated concentration of Sorafenib, EHop-016, or combination therapy for 24 h were measured by flow cytometry. ^*∗*^*P* < 0.05. Statistical differences were analyzed using Student's *t*-tests. Error bars represent SEM from triplicate experiments.

According to the above results, Rac1 could promote sorafenib resistance of HCC cells, we inferred that the Rac1 inhibition could conquer sorafenib resistance. EHop-016 could effectively inhibit the function of Rac1. The decreased sorafenib resistance in hep3B and Huh7 cells was revealed in with EHop-016 treatment by concentration-dependent manner (Figure 5(a)). Then, HCC cells were treated with either sorafenib, EHop-016 (10 *μ*M), or sorafenib and EHop-016 combination for 24 h, and flow cytometry was used to assess the cell cycle and apoptosis level. As shown in Figure 5(b), sorafenib and EHop-016 combination induced a markedly blocked cell cycle. Compared to sorafenib signal therapy, the G2/M cell cycle arrest ratio was significantly upregulated, while sorafenib and EHop-016 combination therapy significantly promoted apoptosis than the control group (Figure 5(c)). Taken together, Rac1 inhibition could reverse the sorafenib chemoresistance.

### 3.6. Rac1 Inhibition Induces Glycolysis Downregulation in HCC Cells

**Figure 6:**
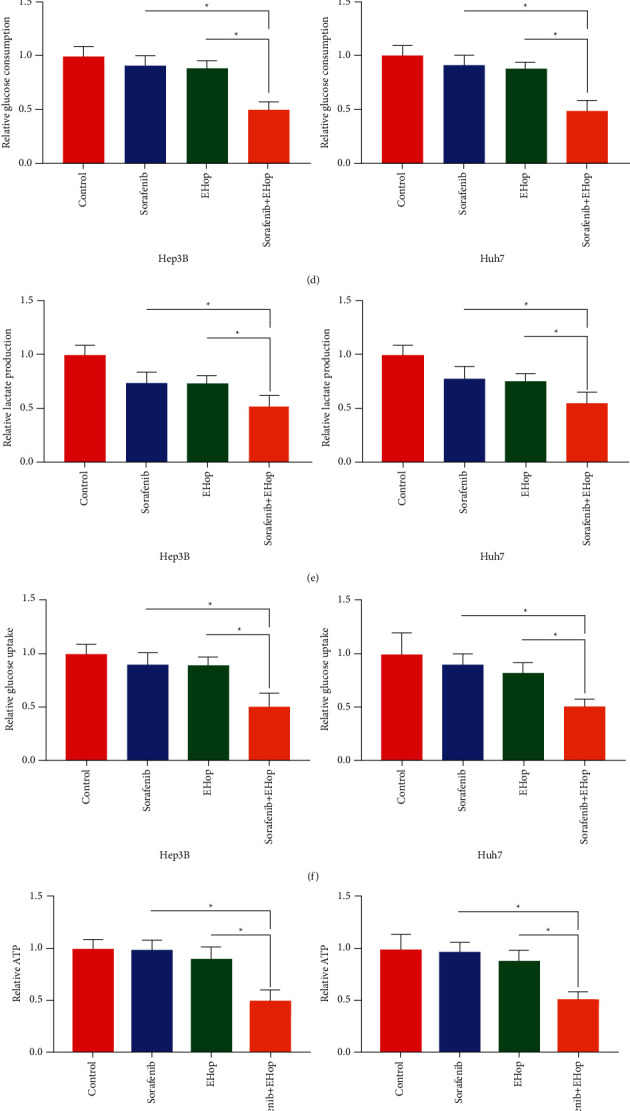
Inhibition of RAC1 blocks glycolysis. (a). RNA-seq analysis represented by a heatmap of gene expression for glycolysis in HCC cells after treatment with Sorafenib, EHop-016 (EHOP), or combination therapy (Sorafenib and EHop) (Figures (b)&(c). The expression of glycolytic enzymes PKM, LDHA, ALDOA, and HK1 was examined using the western blot. (d). Glucose consumption in HCC cells after being treated with Sorafenib, EHop-016, or combination therapy. (e). Lactate production of HCC cells was measured under the treatment of Sorafenib, EHop-016, or combination therapy. (f). Glucose uptake was determined after HCC cells were treated with Sorafenib, EHop-016, or combination therapy. (g). ATP production in HCC cells that were treated with Sorafenib, EHop-016, or combination therapy. ^*∗*^*P* < 0.05. Statistical differences were analyzed using Student's *t*-tests. Error bars represent SEM from triplicate experiments.

In order to detect the underlying mechanisms of the Rac1 inhibitor on chemoresistance of HCC cells to sorafenib, RNA sequencing (RNA-seq) was performed to explore mRNA profiles in different groups. The results revealed that the glycolysis in hep3B cells was significantly inhibited compared with the control group or sorafenib treated (Figure 6(a)). Next, we explored the protein level related to glycolysis. The associated aerobic glycolysis proteins were inhibited by EHop-016 or sorafenib and EHop-016 combination therapy (Figures 6(b) and 6(c)). As shown in Figures 6(d)–6(g), compared with single drug treatment, drug combination markedly prevented glucose consumption (Figure 6(d)), lactate (Figure 6(e)), glucose uptake (Figure 6(f)), and ATP production (Figure 6(g)), were observed in both HCC cells. In summary, Rac1 inhibition could decrease the glycolysis in HCC cells.

### 3.7. Rac1 Inhibition and Sorafenib Combination Prevent Tumor Growth In Vivo

**Figure 7:**
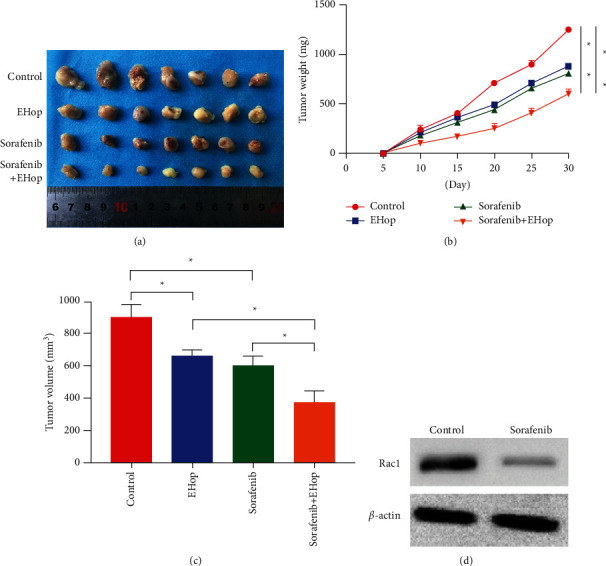
Combining chemotherapy with the RAC1 inhibitor enhances therapeutic effects in HCC xenograft mouse models. (a). Tumors were resected at day 30. (b). Tumor volumes were evaluated every 5 days. (c). Tumor weights were determined at day 30. (d). The expression of RAC1 was detected by the Western blot. ^*∗*^*P* < 0.05. Statistical differences were analyzed using Student's *t*-tests. Error bars represent SEM from triplicate experiments.

Then, Hep3B cells were subcutaneously transplanted to construct hepatocellular carcinoma xenograft models. Sorafenib and EHop-016 combination markedly inhibited tumor growth, and the expression of Rac1 was repressed by sorafenib (Figures 7(a)–7(d)). Taken together, Rac1 inhibition and sorafenib combination prevented tumor growth in vivo.

## 4. Discussion

90% of primary liver cancer is HCC. Because the onset of HCC is hidden, most of the patients have entered the middle and late stage when diagnosed, and they can only be treated with adjuvant comprehensive treatment [26]. However, the emergence of multidrug resistance (MDR) phenomenon in liver cancer chemotherapy greatly reduces the effect of chemotherapy [24]. Therefore, it has become a hot topic for scholars at home and abroad to explore the MDR mechanism of liver cancer and reveal a scheme to reverse liver cancer MDR.

Ras homologue Rho GTP enzyme was previously recognized only for its important role in regulation of actin cytoskeleton. More and more evidences show that the activation of Rho GTP enzyme is associated with oncogenes in many ways. Rho GTP is mainly involved in cell polarization, motility, invasion, proliferation, apoptosis, transcription, cell cycle, cytoskeleton, intercellular adhesion and reactive oxygen species (ROS) product formation, and other physiological activities. Rac1 has attracted wide attention as the most classical member of Rho GTP enzyme. Rac1 is not only involved in cytoskeleton recombination, platypodia formation, and adhesion between normal cells but also closely associated with tumors [27, 28]. The tumor-related studies showed that Rac1 was associated with the occurrence, invasion, apoptosis, and cardiovascular formation of a few tumors. As Rac1 has been studied further, other tumors have been found to be associated with Rac1 abnormalities. Newly discovered tumors (such as cervical cancer) are associated with abnormal Rac1-mediated signaling pathways [29]. Previous studies have shown that some subtypes are associated with abnormal elevated Rac1 expression. At the same time, some of the mechanisms have become clearer, and specific tumors have been identified as being associated with highly active Rac1 mutants. Our research performed that the upregulation of Rac1 in tumor was related to poor prognosis in HCC patients. Furthermore, our vitro data stated that Rac1 overexpression induced HCC cells development, and silencing of Rac1 prevented cell progression. In summary, Rac1 could act as an oncogenic function in HCC progression and development, which could be an underlying biomarker to assess the prognosis of HCC patients.

Sorafenib, as a traditional first-line molecular targeted drug for the therapy of advanced HCC, acts a certain role in the clinical treatment of liver cancer. HCC can participate in sorafenib resistance through autocrine and paracrine pathways and promote the growth and development of HCC. In addition, stromal cells, immune cells, and the extracellular matrix in the tumor microenvironment can also participate in the resistance of HCC to sorafenib through cytokines, hypoxia, and autophagy. The drug resistance caused by long-term oral sorafenib treatment of advanced liver cancer has attracted the attention of scholars all over the world. Understanding the mechanism of drug resistance is helpful for us to find a solution to the drug resistance of sorafenib [30, 31]. Here, our study declared that the Rac1 level is positively associated with sorafenib resistance. Rac1 knockdown could prevent the progression and chemoresistance of HCC cells, and Rac1 knockdown was used by Rac1 inhibitor and EHop-016. Our results indicated that EHop-016 could prevent chemoresistance in vivo and in vitro. Moreover, whether inhibition of Rac1 can not only prevent the drug resistance of sorafenib in liver cancer, but also affect other drug resistance, such as lenvatinib, is needed to investigate in future studies.

Tumor cells supply ATP through the glycolytic pathway. Even in the presence of sufficient oxygen, tumor cells provide energy through the glycolysis pathway. In addition, aerobic glycolysis can produce a large amount of lactic acid, creating an acidic microenvironment for tumor cells, which is conducive to the invasion and metastasis of tumor cells [32]. The changes of glucose metabolism pathway in tumor cells are mainly manifested in increased glucose intake and enhanced glycolytic pathway. Our data demonstrated that glycolytic enzymes were inhibited in the Rac1 inhibitor or combination group. Furthermore, the assay verified similar results of glycolytic enzymes. Furthermore, Rac1 inhibition or the combination group induced glycolysis downregulation.

## 5. Conclusion

Our research reveals that Rac1 induces HCC progression and development and is related to HCC patients' poor prognosis. Rac1 knockdown reverses sorafenib resistance in HCC via glycolysis downregulation. Our results declare the mechanism of Rac1 in regulating HCC cell glycolysis which provides underlying therapeutic target for HCC therapy.

## Data Availability

The datasets used during the present study are available from the corresponding author upon reasonable request.
